# Specific mediator inhibition by the NO donors SNP and NCX 2057 in the peripheral lung: implications for allergen-induced bronchoconstriction

**DOI:** 10.1186/1465-9921-10-46

**Published:** 2009-06-04

**Authors:** Anna-Karin Larsson, Magnus Bäck, Jon O Lundberg, Sven-Erik Dahlén

**Affiliations:** 1 Unit of Lung Biology, Division of Vascular and Respiratory Research, Department of Experimental Medical Science, Lund University, Lund, Sweden; 2 Unit of Experimental Asthma and Allergy Research, Division of Physiology, The National Institute of Environmental Medicine, Karolinska Institutet, Stockholm, Sweden; 3 Department of Cardiology and Center for Molecular Medicine, Karolinska Institutet, Stockholm, Sweden; 4 Department of Physiology and Pharmacology, Karolinska Institutet, Stockholm, Sweden

## Abstract

**Background:**

The aim of this study was to examine potential therapeutic effect of the two NO donors NCX 2057 (3-(4-hydroxy-3-methoxyphenyl)-2-propenoic acid) 4-(nitrooxy)butyl ester) and SNP (sodium nitroprusside) on the early allergic airway response in the peripheral lung.

**Methods:**

The experiments were performed in guinea pig lung parenchyma (GPLP) derived from ovalbumin (OVA) sensitized guinea pigs. The effects of NCX 2057 and SNP were evaluated by contractile responses and mediator release during OVA challenge. The generation of nitrite and nitrate was assessed by chemiluminescence. Statistical analysis was evaluated by ANOVA.

**Results:**

Cumulatively increasing concentrations of OVA (1–10,000 ng/ml) induced concentration-dependent contractions of the GPLP that were reduced by NCX 2057 (100 μM, p < 0.001) and SNP (100 μM, p < 0.05). Antigen-induced eicosanoid release was decreased by NCX 2057 (100 μM, p < 0.001) but not by SNP (100 μM), whereas the release of histamine was reduced by SNP (100 μM, p < 0.001) but not by NCX 2057 (100 μM). In addition, NCX 2057 (0.1–100 μM), but not SNP (0.1–100 μM), relaxed leukotriene D_4 _(10 nM) precontracted GPLP (p < 0.01). The guanylyl cyclase inhibitor ODQ had no effect on the NCX 2057 mediated relaxation. SNP released significantly less nitrite than NCX 2057.

**Conclusion:**

Although both SNP and NCX 2057 reduced the release of pro-inflammatory mediators, their profiles were distinctly different. Furthermore, NCX 2057 also induced smooth muscle dilation in the GPLP. The findings point to specific anti-inflammatory effects of different NO donors in the peripheral lung tissue.

## Background

Administration of exogenous nitric oxide (NO) and nitro vasodilators has received considerable attention, mainly due to their therapeutic ability and haemodynamic effects, and are well established drugs for treatment of cardiac disorders [1,2]. Exogenous NO also has the ability to exert bronchodilatory effects in bronchial asthma [3] and NO is used in the treatment of preterm children to improve lung capacity [4]. However, the effect of NO donors during the early allergic airway response requires further evaluation, especially in the distal lung.

In the peripheral lung, the release of histamine and eicosanoids (leukotrienes and prostaglandins) from activated inflammatory cells, such as mast cells and macrophages, may contribute significantly to the symptoms of allergic rhinitis and asthma [5-8]. In the airways, mast cells and alveolar macrophages also represent a major source of NO [2,9-11], which may act both directly on smooth muscle cells and in an autocrine fashion to suppress allergen-induced responses, as release of histamine [12] and leukotriene synthesis [13]. Inhibitors of NO synthases have been shown to enhance antigen-induced bronchoconstriction in sensitized guinea pigs by increased generation of leukotrienes [14].

**Figure 1:**
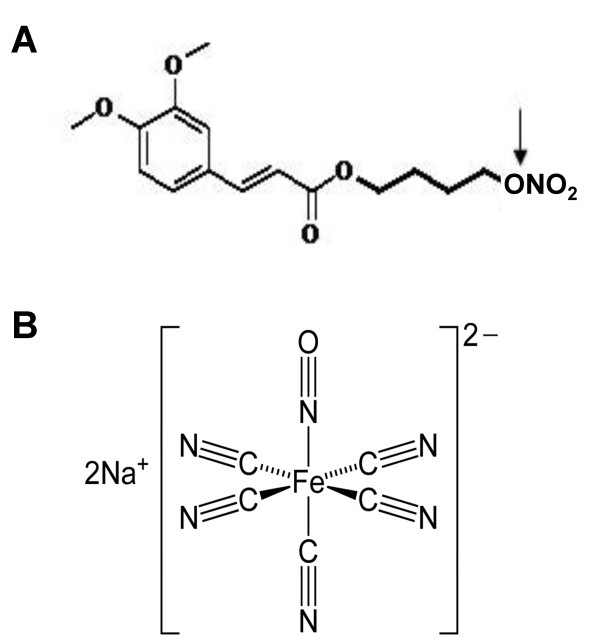
**Chemical structure of the NO donors A, NCX 2057 and B, SNP**. The arrow indicates cleavage site of NO conjugation on substance NCX 2057.

The aim of this study was therefore to examine the role of NO donors in antigen-induced responses in the peripheral part of the lung. Thus, two structurally different NO donors, NCX 2057 (3-(4-Hydroxy-3-methoxyphenyl)-2-propenoic acid) 4-(nitrooxy) butyl ester) [15] and SNP (sodium nitroprusside; Na_2 _[Fe(CN)_5_NO]*2H_2_0) were used (fig 1). The substance NCX 2057 (fig 1a) belongs to a newly class of developed NO donors that are chemically conjugated to a variety of therapeutic drugs, including the anti-histamine cetirizine [15] and NSAIDs [16]. NCX 2057 has also been shown to have anti-inflammatory properties [17]. Therefore, the parent compound of NCX 2057, ferulic acid, was also studied, as this substance has been described to have anti-inflammatory potential [18]. The other NO donor, SNP (fig 1b), is a vasodilator, used in cardiovascular treatments to lower blood pressure or to improve cardiac function [19].

The experiments were performed in actively sensitized guinea pig lung parenchyma (GPLP), an *in vitro *model for antigen-induced contractions [20]. Recent studies of antigen-induced airway constriction in the guinea pig lung indicate that the responses to allergen in humans are similar to the responses obtained in the guinea pig [21,22], since histamine, cysteinyl-leukotrienes (CysLTs) and several prostanoids contribute to the antigen-induced airway constriction in these particular species. The hypothesis to be tested was that NO donors in the peripheral lung may affect antigen-induced contractions either generally by dilatation of smooth muscle tissue or specifically via actions on release of histamine or eicosanoids. The data revealed that the tested NO donors SNP and NCX 2057 acted as inhibitors of allergen-induced mediator release.

## Methods

### Animals and OVA-sensitization

Male Dunkin Hartley guinea pigs (300–350 g b.w.) were sensitized to Chicken Egg Albumin (OVA) at least four weeks prior to experiments [14]. The study was approved by the regional committee of animal experimentation ethics (N127/04).

### Lung parenchymal strips and organ bath experiments

The animals were sacrificed by an overdose of inhaled CO_2 _and the heart-lung-package was quickly removed and placed in ice-cold Tyrode's solution (prepared each day, containing NaCl 149.2 mM, KCl 2.7 mM, NaHCO_3 _11.9 mM, glucose 5.5 mM, CaCl_2 _1.8 mM, MgCl_2 _0.5 mM, NaH_2_PO_4 _0.4 mM). The lung parenchyma was cut parallel to the peripheral margins, yielding four to eight strips, each having a size of 2 × 2 × 20 mm and a weight of approximately 60 mg. The lung parenchymal strips were set up at a resting tension of 2.5 mN (0.25 g) in 5 ml organ baths filled with Tyrode's solution, bubbled with carbogen gas (6.5% CO_2 _in O_2_) to keep a pH of 7.4 and the temperature was kept at 37°C [14,15]. Changes in smooth muscle tension, contractions and relaxations, were recorded via isometric force-displacement transducers connected to a Grass polygraph and responses were displayed by using the IOX data acquisition system (EMKA, France). Data were analyzed by the software program Dataanalyst (EMKA, France). After an equilibration period of 90 min and washes each 15 min, histamine (1–30 μM) was added cumulatively as a control of the GPLP reactivity. Preparations displaying contraction responses less than 1.0 mN to the highest concentration of histamine were excluded from further experiments. Another wash and equilibration period between histamine and treatment period was performed. All drugs were given 15 min before the challenges.

OVA was added as cumulative challenge of increasing concentrations (1–10,000 ng/ml) every 10 min without changing bath fluid. The cumulative OVA concentrations were chosen to generate a concentration-response curve with no contractions at the lowest dose of 1 ng/ml and maximum contractions at the final dose of 10,000 ng/ml OVA. For study of smooth muscle contractions, the GPLP was contracted with cumulative doses of LTD_4 _(0.1–100 nM). 10 nM of LTD4 generated a 50% contraction of the GPLP and this EC_50 _dose was chosen for studies of smooth muscle relaxation. These doses have previously been used in the GPLP *in vitro *test system [14,15,20]. The GPLP was precontracted with LTD_4 _(10 nM) before cumulative addition of cGMP analogue 8-Bromo-cGMP, SNP or NCX 2057. cGMP-dependent relaxation was evaluated by the addition of the guanylyl cyclase inhibitor ODQ (30 μM), which previously has been demonstrated to inhibit vasodilation induced by NCX 2057 [15]. Maximum contractions of the preparation were determined with histamine (1 mM), acetylcholine (1 mM) and KCl (50 mM) at the end of each experiment, and other responses were expressed as percent of maximum contractions.

### Measurements of released mediators with enzyme immunoassays

A 1-mL aliquot of organ bath fluid was collected from each organ bath and immediately frozen at -20°C. The samples were taken at the end of the equilibration period to obtain basal mediator release from the tissue and at the obtained contractile plateau after challenge with OVA 1000 ng/ml or at the obtained contractile plateaus after cumulative doses of OVA (1, 10, 100 and 1000 ng/ml). Enzyme immunoassay (EIA) analyses of the different mediators LTB_4_, CysLTs and the prostanoids thromboxane (TXA_2_) and prostaglandin (PG) D_2 _were performed according to the manufacture's instructions. TXA_2 _was measured as the stable metabolite TXB_2_. PGD_2 _was measured as PGD_2_-mox. The assay detection limits in the bath fluid levels for the different mediators were 7.8 pg/ml for TXB_2_, PGD_2 _and LTE_4 _and 3.9 pg/ml for LTB_4_. Results below detection limits were set as zero in the statistical evaluation. The EIA specificity for the different mediators to interfere with each other was less than 0.01%, with the exception of the EIA kit for TXB_2 _cross reacted with PGD_2 _(0.53%) and with PGE_2 _(0.09%). The EIA kit for CysLTs was performed with the CysLT antiserum and the sera cross reacted with LTC_4 _(100%) and LTD_4 _(100%) and LTE_4 _(67%). Histamine was measured as previously described [23,24]. Duplicates of 300 μl were placed in 96-wells plates and the amount of histamine was analyzed by a fluorospectrometer at the wavelength 450 nM. The detection limit for histamine was 3.9 ng/ml.

### Measurements of nitrite and nitrate with chemiluminescence

Aliquots of bath fluid were collected from the organ bath for measurements of nitrite and nitrate to obtain an indirect assessment of the NO releasing profile for the NO donors. To determine the basal production of nitrite and nitrate in the bath fluid, a sample volume of 200 μl was withdrawn from each bath before the addition of any drug. Bath fluid aliquots (200 μl) were then collected at 15, 30, 60, 90 and 120 min after the addition of Tyrode's solution, DMSO, NCX 2057 (100 μM) or SNP (100 μM). The amount of nitrite and nitrate was analyzed with a chemiluminescence method as previously described [25].

### Data analysis and statistical procedures

All data are presented as mean ± standard error of the mean (s.e.m.). Statistical analyses were made for paired and unpaired observations by Student's t-test or analyses of variances (ANOVA). All concentration-response curves were statistically analyzed with two-way ANOVA followed by the post hoc tests Tukey's t-test or Bonferroni's t-test. A p-value of less than 0.05 was considered significant.

### Drugs and chemical reagents

NaCl, KCl, CaCl_2_, MgSO_4_, NaHCO_3_, KH_2_PO_4 _and glucose were obtained from VWR International (West Chester, Pennsylvania, USA). Histamine dihydrochloride, acetylcholine, ovalbumin (OVA, chicken egg albumin, grade II), dimethylsulfoxid (DMSO), ferulic acid, ODQ (1H(1,2,4)oxadiazolo(4,3-a)quinoxalin-1-one), 8-bromo-cGMP and SNP were purchased from Sigma-Aldrich (St. Louis, Missouri, USA). LTD_4 _was from Cascade Biochemicals Ltd. (Reading, UK). The EIA kits for CysLTs, LTB_4_, TXB_2 _and PGD_2_-mox were obtained from Cayman Chemicals (Ann Arbor, Michigan, USA). NCX 2057 (3-(4-hydroxy-3 methoxyphenyl)-2-propenoic acid 4-(nitroxy) butyl ester) was a kind gift from NicOx (Bresso, Milan, Italy) and Biolipox (Stockholm, Sweden). Stock solutions of 1 mM LTD_4 _were dissolved in 50% ethanol-water and then diluted in 20% ethanol-water. The concentration and purity of LTD_4 _was checked by UV spectroscopy. NCX 2057 and ferulic acid were dissolved in DMSO. OVA was dissolved in 0.9% NaCl. The other drugs were dissolved and diluted in Tyrode's solution or millipure water. Dilutions of drugs were freshly made from the stocks for each experiment. The drugs were present in the organ bath fluid during the remaining experiment. 5 μl of DMSO was added as a control and did not influence the baseline or cumulative contractions to OVA.

## Results

### Effect of SNP and NCX 2057 on antigen-induced contractions

**Figure 2:**
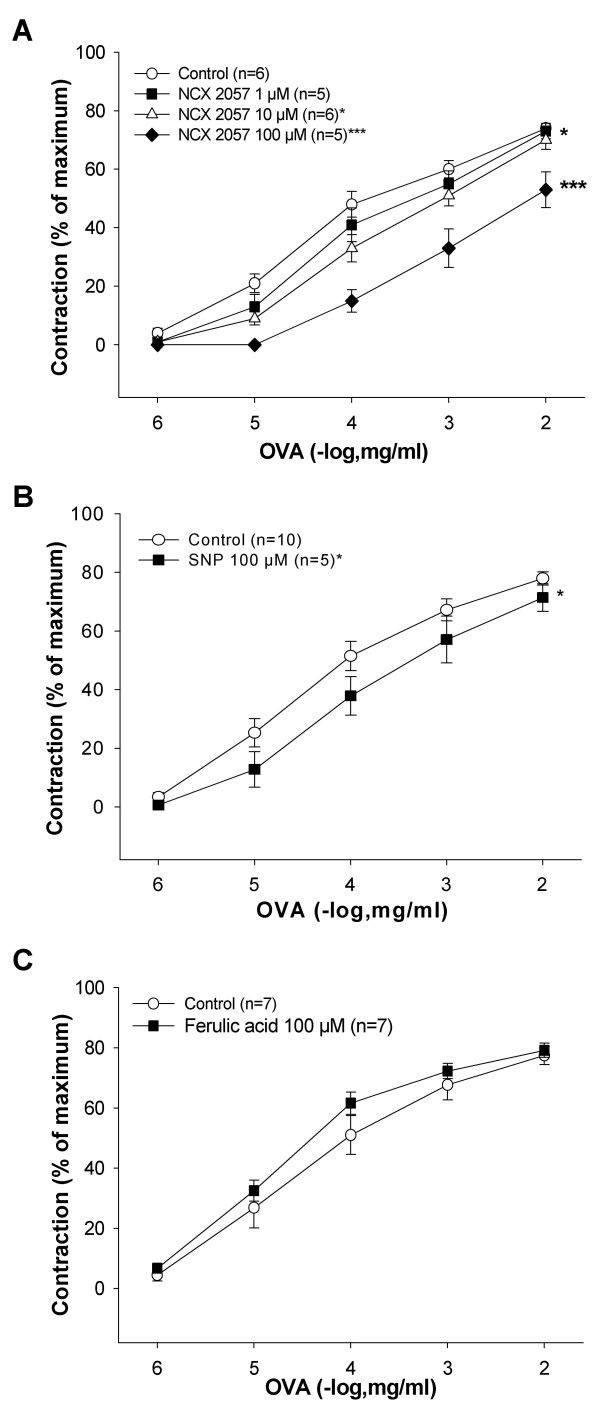
**Drug effects on the concentration-response to OVA (1–10,000 ng/ml) in sensitized lung parenchymal strips**. A, effect of pretreatment with NCX 2057 (1 μM; n = 5), NCX 2057 (10 μM; n = 6) and NCX 2057 (100 μM; n = 5) compared to control (n = 6). B, effect of pretreatment with SNP (100 μM; n = 5) compared to control (n = 10). C, effect of pretreatment with 100 μM ferulic acid (n = 7) compared to control (n = 7). Data are expressed as mean ± s.e.m.; statistical analysis was performed by two-way ANOVA. *P < 0.05; ***P < 0.001 *vs *control.

Cumulative doses of OVA (1–10,000 ng/ml) induced concentration-dependent contractions of the GPLP. Pretreatment with NCX 2057 (1, 10 and 100 μM) concentration-dependently inhibited the response to OVA (p < 0.05, p < 0.001; fig 2a). Pretreatment with SNP (100 μM) attenuated the contractions to OVA (p < 0.05, fig 2b). Ferulic acid (100 μM) had no significant effect on the OVA response (fig 2c).

### Effect on the release of mediators upon ovalbumin challenge

**Figure 3:**
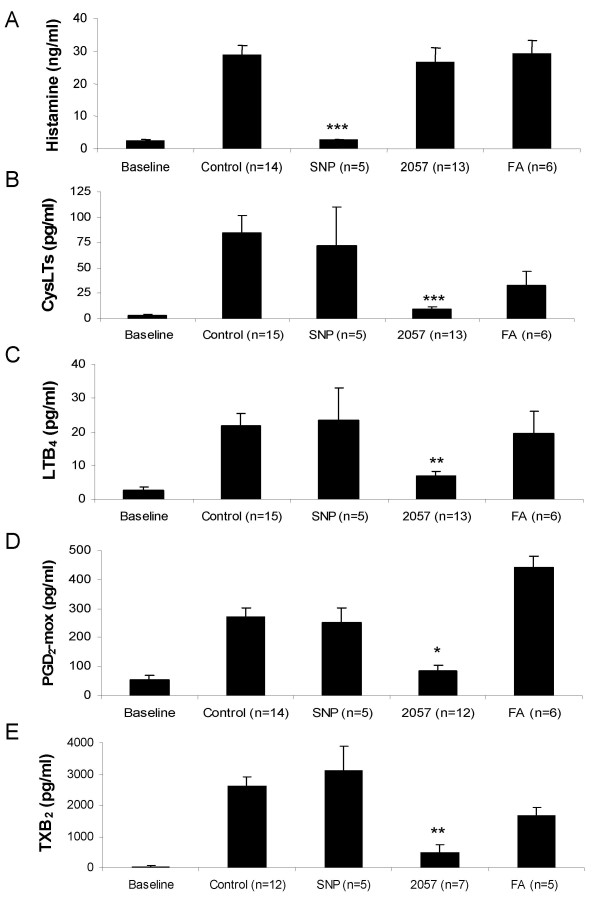
**Effect of pretreatment with different drugs compared with the control on mediator release from sensitized GPLP after challenge with 1000 ng/ml of OVA**. Release of A, Histamine (ng/ml); B, CysLTs; C, LTB_4_; D, PGD_2_; E, TXB_2 _(pg/ml). All samples were collected at baseline and then at the plateau after 1000 ng/ml of OVA. The parenchymal strips had been pretreated for 15 min with Tyrode's solution (control), 100 μM SNP (SNP), 100 μM NCX 2057 (2057) and 100 μM ferulic acid (FA). All data are expressed as mean ± s.e.m.; statistical analysis was performed by one-way ANOVA. *, P < 0.05; **, P < 0.01; ***, P < 0.001 *vs *control.

**Figure 4:**
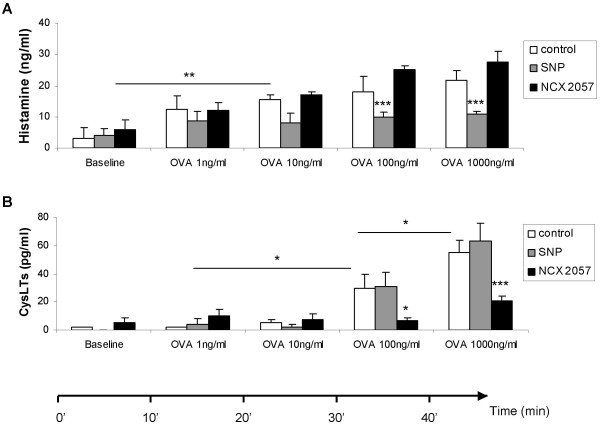
**Effect of pretreatment with SNP (100 μM, n = 4, grey) and NCX 2057 (100 μM, n = 4, black) compared to control (Tyrode's solution, n = 4, white) on mediator release from sensitized GPLP after cumulative challenge with OVA**. A, Histamine (ng/ml). B, CysLTs (pg/ml). All samples were collected at baseline and then after 10 min at the plateau of cumulative addition of 1, 10, 100 and 1000 ng/ml OVA. All data are expressed as mean ± s.e.m.; statistical analysis was performed by two-way ANOVA.*P < 0.05; ***P < 0.001 *vs *control.

The mediator release from the lung tissue was analyzed in the bath fluid to further describe the inhibitory effects of SNP and NCX 2057 on the OVA-induced contractions. Basal release in the bath fluid before OVA challenge was below detection limit for histamine, CysLTs and LTB_4_, whereas measurable levels were obtained for TXB_2 _(46 ± 11 pg/ml) and PGD_2 _(56 ± 16 pg/ml) (fig 3). After challenge with OVA (1000 ng/ml) measurable levels were obtained for all the mediators with the rank order of histamine >> TXB_2 _> PGD_2 _> CysLTs > LTB_4 _(fig 2). SNP (100 μM) significantly reduced the release of histamine (p < 0.001, fig 3a), but did not affect the release of eicosanoids. NCX 2057 (100 μM) did not change the release of histamine but significantly inhibited the release of eicosanoids; LTB_4 _(p < 0.001, fig 3b), CysLTs (p < 0.001, fig 3c), PGD_2 _(p < 0.05, fig 3d) and TXB_2 _(p < 0.01, fig 3e). Ferulic acid (100 μM) had no significant effects on the measured mediators after challenge with OVA 1000 ng/ml (fig 3). To see any distinction in the release profile between histamine and CysLTs the bath fluid was analysed for all the cumulative doses of OVA (1–1000 ng/ml). Histamine was released at low doses of OVA and did not increase significantly at higher doses of OVA (fig 4a). In contrast, CysLTs were dose-dependently generated after cumulative administration of OVA (fig 4b). The release of histamine was significantly inhibited by SNP (100 μM, p < 0.001), whereas NCX 2057 (100 μM) did not significantly affect this release (fig 4a). The synthesis of CysLTs was significantly inhibited by NCX 2057 (100 μM, p < 0.001) after cumulative administration of OVA (fig 4b).

### Analysis of nitrate and nitrite generation from SNP and NCX 2057

**Figure 5:**
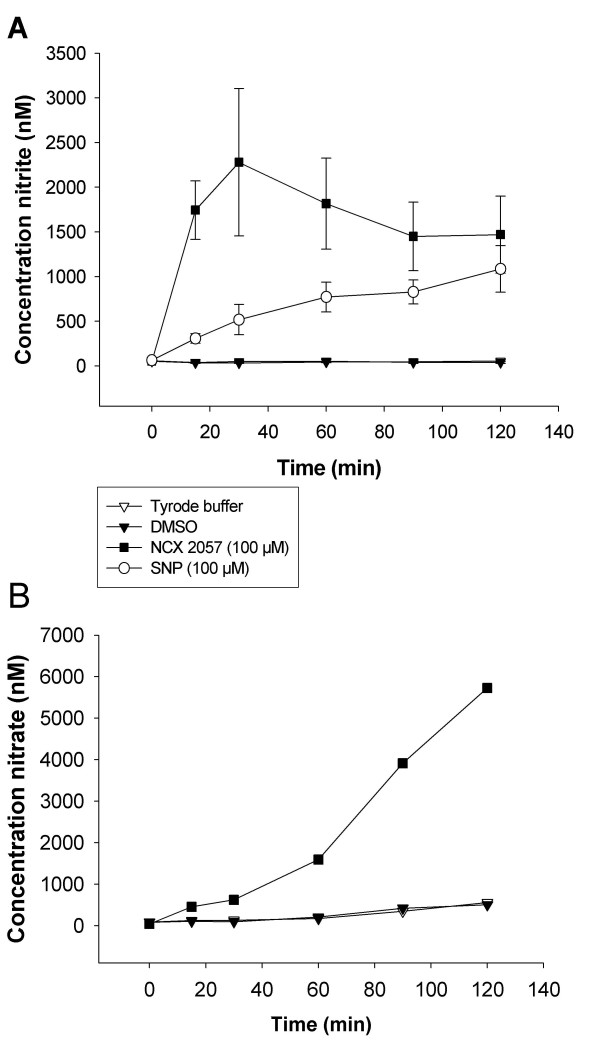
**A, Generation of nitrite (nM) over two hours in the GPLP organ bath fluid after addition of SNP (100 μM; n = 3), NCX 2057 (100 μM; n = 3), DMSO (n = 3) or Tyrode's buffer (n = 3)**. **B**, Generation of nitrate over two hours in the GPLP organ bath fluid after addition of NCX 2057 (100 μM; n = 3), DMSO (n = 3) or Tyrode's buffer (n = 3). Data are expressed as mean ± s.e.m.

NCX 2057 (100 μM, n = 3) generated nitrite with a peak of 2280 ± 824 nM after 30 min. The nitrite generation was at one hour replaced by the generation of nitrate (1597 ± 345 nM) that continuously increased to 5729 ± 466 nM at two hours (fig 5). SNP (100 μM; n = 3) continuously released nitrite at around 800 nM for two hours in the organ bath (fig 5). The generation of nitrate from SNP was not possible to assess due to unspecific binding during analysis. The solvent DMSO (5 μl) or Tyrode's buffer did not generate any nitrite or nitrate (n = 3, fig 5).

### Effect as smooth muscle dilators on LTD_4_-contracted lung parenchyma

**Figure 6:**
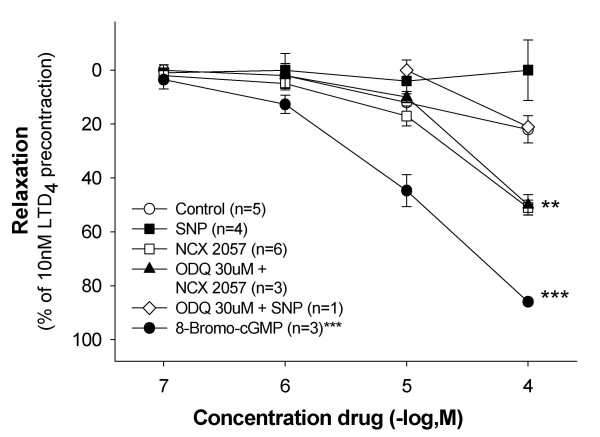
**Effect of cumulative concentrations of 8-bromo-cGMP (0.1–100 μM, n = 3), SNP (0.1–100 μM, n = 4), NCX 2057 (0.1–100 μM) alone (n = 6) and in combination with 30 μM ODQ (n = 3) compared to control (n = 5) on contractions induced by 10 nM of LTD_4 _on GPLP**. Data are expressed as mean ± s.e.m.; statistical analysis was performed by two-way ANOVA. *P < 0.05;^##^, **P < 0.01; ***P < 0.001 *vs *control.

**Figure 7:**
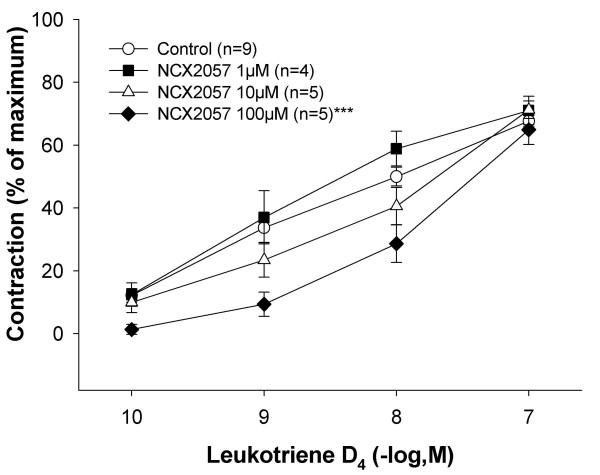
**Effect of pretreatment with NCX 2057 (1 μM, n = 4; 10 μM, n = 5; 100 μM, n = 5) on the concentration-response to LTD_4 _(0.1–100 nM) in GPLP compared to control (n = 9)**. All data are expressed as mean ± s.e.m.; statistical analysis was performed by two-way ANOVA. ***P < 0.001 *vs *control.

SNP (0.1–100 μM) displayed no relaxant effect on the LTD_4_-induced precontraction, whereas NCX 2057 (0.1–100 μM) relaxed the lung parenchyma at higher concentrations (100 μM, p < 0.01). The cGMP analogue 8-Bromo-cGMP (0.1–100 μM) concentration-dependently relaxed the GPLP compared to control (Tyrode's solution) (p < 0.001). Pretreatment with the guanylyl cyclase inhibitor ODQ (30 μM) did not affect the response to either SNP (1–100 μM, data not shown) or NCX 2057 (1–100 μM) (fig 6). NCX 2057 (1–100 μM) attenuated the contractions to cumulative doses of LTD_4 _(0.1–100 nM) at the dose 100 μM (p < 0.001, fig 7). Lower doses of NCX 2057 had no effect on the LTD_4_-induced contractions.

## Discussion

In this study we evaluated the effect of SNP and NCX 2057 during antigen-induced contractions in the peripheral lung. Our data indicated that the tested NO donors acted specifically on the release of different antigen-induced mediators and also differently as smooth muscle dilators. These data imply that depending on the nature of the NO donor, specific inhibition of antigen-induced contraction occurs and results in consistent inhibition of contraction in response to antigen.

Recent studies of antigen-induced airway constriction in the guinea pig lung indicate that histamine, CysLTs and several prostanoids, as PGD_2 _and TXA_2_, contribute to the antigen-induced airway constriction [20-22]. Histamine is released from granulars in mast cells and basophils and histamine is an important mediator of the early allergic airway response and airway inflammation in asthma [5]. Leukotrienes are biosynthesised *de novo *via 5-lipoxygenase from cell membrane phospholipids in mast cells, eosinophils, neutrophils, basophils and alveolar macrophages [26]. Prostanoids are generated *de novo *by cyclooxygenase (COX) enzymes in most cells [7]. However, PGD_2 _is mainly produced by mast cells and is known as a marker of mast cell activation [27] and associated with inflammatory conditions [28]. PGD_2 _is released in the absence of degranulation, and may be stored or rapidly synthesized and released in distinct pathways from degranulation after IgE ligation. TXA_2 _is mainly produced by platelets, macrophages and neutrophils [7]. TXA_2 _is increased in the airways of asthmatics after allergen challenge [6]. In the present study, measurements of histamine and eicosanoids in the bath fluid after challenges with antigen showed that histamine was released in highest amounts followed by prostanoids and then leukotrienes from the GPLP preparations. However, leukotrienes are known to be 1000 times more potent bronchoconstrictors than histamine and the induced contractions are more long lasting [29]. Interestingly, NCX 2057 and SNP had different inhibitory profile on the measured mediators in the present study. SNP blocked the release of histamine after challenge with OVA, whereas NCX 2057 reduced the synthesis of leukotrienes and prostanoids, suggesting that SNP and NCX 2057 may attenuate the activation of inflammatory cells and mediator release in distinctly different steps. NOx-containing molecules may react with oxygen, superoxides, water, nucleotides, metalloproteins, thiols, amines, and lipids to form products with biochemical actions [2] in the peripheral lung tissue. NO may also bind to iron-containing enzymes via nitrosylation and form iron-nitrosyl complexes, resulting in altered enzymatic activity and function [30]. S-nitrosylation is an important posttranslational modification that regulates NO transport and cell signalling, and thereby the activity of a vast number of proteins in different cell types, including mast cells and macrophages [30-32], and may be a crucial regulator of airway responsiveness [33]. There are implications that NO decrease leukotriene synthesis in mast cells [13] and macrophages [11]. A possible explanation of the inhibitory effects of NCX2057 in the present study, is therefore that this lipophilic NO donor has the potential to inhibit *de novo *synthesis of leukotrienes and prostanoids via interaction with iron centre in 5-lipoxygenase and cyclooxygenase enzymes [34,35], and via interaction with thiol groups on LTC_4 _synthase [34]. In a previous study in the GPLP *in vitro *system, inhibition of NO synthases enhanced antigen-induced contractions by increasing the synthesis of CysLTs in the peripheral lung [14]. The effect of NCX 2057 on the antigen-induced response could also be explained by the fact that this molecule is derived from the parent compound ferulic acid, a substance described to have anti-inflammatory and anti-oxidant potentials [18,36]. NCX 2057 has previously been implicated to have anti-inflammatory properties [17]. Nonetheless, in the present study the ferulic acid substance did not influence either the antigen-induced contractions or the release of pro-inflammatory mediators, suggesting that the inhibitory effect of NCX 2057 is due to its NO conjugation. The other tested NO donor, SNP, completely reduced the release of histamine after OVA challenge in this study. Topical administration of SNP has previously been shown to reduce histamine-induced plasma exudation both in guinea pig *in vivo *and human nasal airways [37] and previous findings in the rat mast cell confirm that both SNP and nitrite may inhibit the release of histamine [38,39], suggesting that SNP has a specific mode of action on degranulation. SNP was over all less effective than NCX 2057 in this study. Previous studies in the GPLP *in vitro *system show that antagonists to the histamine receptor, such as cetirizine, has very little effect on the early allergic response in the peripheral lung [15]. This is in line with the results shown by SNP as a potential histamine inhibitor in these experiments.

SNP did not relax the lung parenchyma whereas higher concentrations of NCX 2057 (100 μM) induced relaxation of the smooth muscle tissue in the present study. These results are in contrast to the previous findings in guinea pig aorta and pulmonary artery where NCX 2057 induced vasodilation at much lower concentrations (nM) [15], supporting the hypothesis that the smooth muscle tissue in the peripheral lung is less sensitive to NO mediated dilations. The guanylyl cyclase inhibitor ODQ did not inhibit the relaxation induced by NCX 2057 despite the fact that 8-Bromo-cGMP induced strong relaxation in the GPLP system. This is in contrast to the cGMP-dependent dilation shown by NCX 2057 in vascular system in a previous study where ODQ effectively prevented the induced vasodilation [15]. Nevertheless, nitrovasodilators have previously been shown to induce smooth muscle relaxation both dependent and independent via cGMP activation [40-42].

Although the tested substances are referred as NO donors, the effect of SNP and NCX 2057 in the peripheral lung may not only depend on NO generation. The concentrations of the pharmacological agents were higher than are ordinarily used *in vivo *and also higher than NO levels produced *in vivo*. Measurements of nitrite and nitrate indicated that SNP and NCX 2057 differed notably in their kinetic profile. NCX 2057 produced significantly more nitrite than SNP at early timepoints (fig. 4). Nitrite accumulation is generally interpreted as an indicator of NO release. Thus, this could be a possible explanation for the decreased effectiveness of SNP relative to NCX 2057 as an inhibitor of lung contractility. However, it is important to realize that drugs may be metabolised to nitrate and nitrite without apparent formation of NO and therefore one cannot rely completely of nitrate/nitrite measurements as accurate estimates of NO formation. Several additional factors determine the rate of NO release and bioavailability of different NO donors including, lipophilicity, site of NO release, the presence of metal containing and thiol containing compounds, the stability of the NO bonds, and the propensity for homolytic vs. heterolytic cleavage [43]. In contrast to NCX 2057, SNP also transfers NO^+ ^[43,44], which can react with sulphydryl groups (-SH), to form S-nitrosothiols [45]. The mechanisms behind the distinct inhibitory effects of the tested NO donors on the early allergic airway response need to be further evaluated in other test systems.

## Conclusion

The obtained results point at important differences between the tested NO donors, making it crucial to carefully characterize the profile of a selected NO donor, when using it as an experimental tool. The data revealed that the tested NO donors SNP and NCX 2057 acted as specific inhibitors of allergen-induced mediator release. In contrast to SNP, NCX 2057 also induced relaxation in the GPLP, but not via the cGMP pathway. The findings support that different NO donors may have specific anti-inflammatory effects in the peripheral lung tissue and may facilitate the development of anti-inflammatory therapeutic strategies targeting distinct effects of early allergic airway response.

## Abbreviations

EIA: enzyme immuno assay; GPLP: guinea pig lung parenchyma; LT: leukotriene; NCX 2057: 3-(4-hydroxy-3 methoxyphenyl)-2-propenoic acid 4-(nitroxy) butyl ester; OVA: ovalbumin; PG: prostaglandin; SNP: sodium nitroprusside.

## Competing interests

The authors declare that they have no competing interests.

## Authors' contributions

AKL conceived and participated in the design of the study, carried out the study, performed the statistical analysis, interpretation of data and drafted the manuscript. MB conceived and participated in the design of the study, interpretation of data and helped to draft the manuscript. JL participated in the design, interpretation of data and helped to draft the manuscript. SED conceived and participated in the design of the study, interpretation of data and helped to draft the manuscript. All authors read and approved the final manuscript.

## References

[B1] Rastaldo R, Pagliaro P, Cappello S, Penna C, Mancardi D, Westerhof N, Losano G (2007). Nitric oxide and cardiac function. Life Sci.

[B2] Gaston B, Drazen JM, Loscalzo J, Stamler JS (1994). The biology of nitrogen oxides in the airways. Am J Respir Crit Care Med.

[B3] Hogman M, Frostell CG, Hedenstrom H, Hedenstierna G (1993). Inhalation of nitric oxide modulates adult human bronchial tone. Am Rev Respir Dis.

[B4] Barrington KJ, Finer NN (2007). Inhaled nitric oxide for respiratory failure in preterm infants. Cochrane Database Syst Rev.

[B5] Lordan JL, Holgate ST (2002). H1-antihistamines in asthma. Clin Allergy Immunol.

[B6] Wenzel SE, Westcott JY, Larsen GL (1991). Bronchoalveolar lavage fluid mediator levels 5 minutes after allergen challenge in atopic subjects with asthma: relationship to the development of late asthmatic responses. J Allergy Clin Immunol.

[B7] Rolin S, Masereel B, Dogne JM (2006). Prostanoids as pharmacological targets in COPD and asthma. Eur J Pharmacol.

[B8] Dahlen SE (2006). Treatment of asthma with antileukotrienes: first line or last resort therapy?. Eur J Pharmacol.

[B9] Gilchrist M, Savoie M, Nohara O, Wills FL, Wallace JL, Befus AD (2002). Nitric oxide synthase and nitric oxide production in in vivo-derived mast cells. J Leukoc Biol.

[B10] Dean Befus YS, Moon TC, Munoz S, Befus AD (2005). Role of nitric oxide in mast cells: controversies, current knowledge, and future applications. Immunol Res.

[B11] Coffey MJ, Phare SM, Peters-Golden M (2000). Prolonged exposure to lipopolysaccharide inhibits macrophage 5-lipoxygenase metabolism via induction of nitric oxide synthesis. J Immunol.

[B12] Masini E, Salvemini D, Pistelli A, Mannaioni PF, Vane JR (1991). Rat mast cells synthesize a nitric oxide like-factor which modulates the release of histamine. Agents Actions.

[B13] Gilchrist M, McCauley SD, Befus AD (2004). Expression, localization, and regulation of NOS in human mast cell lines: effects on leukotriene production. Blood.

[B14] Larsson AK, Back M, Hjoberg J, Dahlen SE (2005). Inhibition of nitric-oxide synthase enhances antigen-induced contractions and increases release of cysteinyl-leukotrienes in guinea pig lung parenchyma: nitric oxide as a protective factor. J Pharmacol Exp Ther.

[B15] Larsson AK, Fumagalli F, DiGennaro A, Andersson M, Lundberg J, Edenius C, Govoni M, Monopoli A, Sala A, Dahlen SE, Folco GC (2007). A new class of nitric oxide-releasing derivatives of cetirizine; pharmacological profile in vascular and airway smooth muscle preparations. Br J Pharmacol.

[B16] Fiorucci S, Antonelli E (2006). NO-NSAIDs: from inflammatory mediators to clinical readouts. Inflamm Allergy Drug Targets.

[B17] Wenk GL, McGann-Gramling K, Hauss-Wegrzyniak B, Ronchetti D, Maucci R, Rosi S, Gasparini L, Ongini E (2004). Attenuation of chronic neuroinflammation by a nitric oxide-releasing derivative of the antioxidant ferulic acid. J Neurochem.

[B18] Graf E (1992). Antioxidant potential of ferulic acid. Free Radic Biol Med.

[B19] Shin DD, Brandimarte F, De Luca L, Sabbah HN, Fonarow GC, Filippatos G, Komajda M, Gheorghiade M (2007). Review of current and investigational pharmacologic agents for acute heart failure syndromes. Am J Cardiol.

[B20] Jonsson EW, Dahlen SE (1994). Interactions between leukotrienes and histamine in the anaphylactic contraction of guinea pig lung parenchyma. J Pharmacol Exp Ther.

[B21] Ressmeyer AR, Larsson AK, Vollmer E, Dahlen SE, Uhlig S, Martin C (2006). Characterisation of guinea pig precision-cut lung slices: comparison with human tissues. Eur Respir J.

[B22] Sundstrom E, Lastbom L, Ryrfeldt A, Dahlen SE (2003). Interactions among three classes of mediators explain antigen-induced bronchoconstriction in the isolated perfused and ventilated guinea pig lung. J Pharmacol Exp Ther.

[B23] Shore PA, Burkhalter A, Cohn VH (1959). A method for the fluorometric assay of histamine in tissues. J Pharmacol Exp Ther.

[B24] Bergendorff A, Uvnas B (1972). Storage of 5-hydroxytryptamine in rat mast cells. Evidence for an ionic binding to carboxyl groups in a granule heparin-protein complex. Acta Physiol Scand.

[B25] Lundberg JO, Govoni M (2004). Inorganic nitrate is a possible source for systemic generation of nitric oxide. Free Radic Biol Med.

[B26] Kanaoka Y, Boyce JA (2004). Cysteinyl leukotrienes and their receptors: cellular distribution and function in immune and inflammatory responses. J Immunol.

[B27] Dahlen SE, Kumlin M (2004). Monitoring mast cell activation by prostaglandin D2 in vivo. Thorax.

[B28] LJ Roberts, Sweetman BJ, Lewis RA, Austen KF, Oates JA (1980). Increased production of prostaglandin D2 in patients with systemic mastocytosis. N Engl J Med.

[B29] Dahlen SE, Hedqvist P, Westlund P, Granstrom E, Hammarstrom S, Lindgren JA, Radmark O (1983). Mechanisms of leukotriene-induced contractions of guinea pig airways: leukotriene C4 has a potent direct action whereas leukotriene B4 acts indirectly. Acta Physiol Scand.

[B30] Lancaster JR, Langrehr JM, Bergonia HA, Murase N, Simmons RL, Hoffman RA (1992). EPR detection of heme and nonheme iron-containing protein nitrosylation by nitric oxide during rejection of rat heart allograft. J Biol Chem.

[B31] Forsythe P, Befus AD (2003). Inhibition of calpain is a component of nitric oxide-induced down-regulation of human mast cell adhesion. J Immunol.

[B32] Lim SY, Raftery M, Cai H, Hsu K, Yan WX, Hseih HL, Watts RN, Richardson D, Thomas S, Perry M, Geczy CL (2008). S-nitrosylated S100A8: novel anti-inflammatory properties. J Immunol.

[B33] Que LG, Liu L, Yan Y, Whitehead GS, Gavett SH, Schwartz DA, Stamler JS (2005). Protection from experimental asthma by an endogenous bronchodilator. Science.

[B34] Coffey MJ, Coles B, O'Donnell VB (2001). Interactions of nitric oxide-derived reactive nitrogen species with peroxidases and lipoxygenases. Free Radic Res.

[B35] Goodwin DC, Landino LM, Marnett LJ (1999). Effects of nitric oxide and nitric oxide-derived species on prostaglandin endoperoxide synthase and prostaglandin biosynthesis. Faseb J.

[B36] Hosoda A, Ozaki Y, Kashiwada A, Mutoh M, Wakabayashi K, Mizuno K, Nomura E, Taniguchi H (2002). Syntheses of ferulic acid derivatives and their suppressive effects on cyclooxygenase-2 promoter activity. Bioorg Med Chem.

[B37] Greiff L, Andersson M, Svensson C, Nilsson M, Erjefalt I, Erjefalt JS, Persson CG (1995). Topical nitroprusside may reduce histamine-induced plasma exudation in human nasal airways. Allergy.

[B38] Masini E, Di Bello MG, Pistelli A, Raspanti S, Gambassi F, Mugnai L, Lupini M, Mannaioni PF (1994). Generation of nitric oxide from nitrovasodilators modulates the release of histamine from mast cells. J Physiol Pharmacol.

[B39] Mannaioni PF, Bello MG, Di Bello MG, Mirabella C, Gai P, Schunack W, Masini E (1997). Interaction between histamine and nitric oxide in rat mast cells and in isolated guinea pig hearts. Int Arch Allergy Immunol.

[B40] Branka JE, Vallette G, Jarry A, Laboisse CL (1997). Stimulation of mucin exocytosis from human epithelial cells by nitric oxide: evidence for a cGMP-dependent and a cGMP-independent pathway. Biochem J.

[B41] Jansen A, Drazen J, Osborne JA, Brown R, Loscalzo J, Stamler JS (1992). The relaxant properties in guinea pig airways of S-nitrosothiols. J Pharmacol Exp Ther.

[B42] Gaston B, Drazen JM, Jansen A, Sugarbaker DA, Loscalzo J, Richards W, Stamler JS (1994). Relaxation of human bronchial smooth muscle by S-nitrosothiols in vitro. J Pharmacol Exp Ther.

[B43] Feelisch M (1998). The use of nitric oxide donors in pharmacological studies. Naunyn Schmiedebergs Arch Pharmacol.

[B44] Govoni M, Casagrande S, Maucci R, Chiroli V, Tocchetti P (2006). In vitro metabolism of (nitrooxy)butyl ester nitric oxide-releasing compounds: comparison with glyceryl trinitrate. J Pharmacol Exp Ther.

[B45] Gaston B, Singel D, Doctor A, Stamler JS (2006). S-nitrosothiol signaling in respiratory biology. Am J Respir Crit Care Med.

